# Our Contemporaries

**Published:** 1914-08

**Authors:** 


### A Matter of Business.

Not including bills for the volume just beginning, the total of subscriptions in arrears exceed $1200. This is enough to pay for three or four issues of the JOURNAL. In spite of the hard times, the JOURNAL is thriving and has maintained more advertising than any other medical journal in the country of comparable scope, with possibly two or three exceptions. But while no periodical, save a few published at high subscription rates without advertising, regards its subscriptions as a major asset, a large number of small bills outstanding means a serious hardship. Our readers know how this is themselves, from their professional experience. There is this difference: We practice medicine to earn our living and because, on the whole, we would rather do that than anything else, even than leading the life of a gentleman, as it is cynically defined. The editorship of a journal is rather a self-imposed matter of public spirit than one of business although, under normal conditions, it should not involve direct expense and should even pay a small sum for the time and and labor expended, with due regard to the fact that such labòr is along general professional lines of interest.

It is distasteful to dun professional colleagues but we want to remind them that a prosperous journal means a good journal and that every minute which has to be devoted to sordid business details detracts so much from the necessarily limited time that can be devoted to the literary and scientific interests of the journal.

SUBSCRIPTION BILLS ARE INCLOSED WITH THIS ISSUE. As a favor to us, please report any error of address or bookkeeping promptly.

---

